# A Traditional Review of Sickle Cell Disease and the Associated Onset of Dementia: Hematological and Neurocognitive Crossroads

**DOI:** 10.7759/cureus.18906

**Published:** 2021-10-19

**Authors:** Ugochi Ojinnaka, Zubayer Ahmed, Amudhan Kannan, Huma Quadir, Knkush Hakobyan, Mrunanjali Gaddam, Jihan A Mostafa

**Affiliations:** 1 Family Medicine, California Institute of Behavioral Neurosciences & Psychology, Fairfield, USA; 2 Internal Medicine, California Institute of Behavioral Neurosciences & Psychology, Fairfield, USA; 3 Medicine, Jawaharlal Institute of Postgraduate Medical Education and Research, Puducherry, IND; 4 General Surgery, Research, California Institute of Behavioral Neurosciences & Psychology, Fairfield, USA; 5 Internal Medicine, Family Medicine, Neurology, California Institute of Behavioral Neurosciences & Psychology, Fairfield, USA; 6 Diagnostic Radiology, California Institute of Behavioral Neurosciences & Psychology, Fairfield, USA; 7 Psychiatry, California Institute of Behavioral Neurosciences & Psychology, Fairfield, USA

**Keywords:** sickle cell disease, dementia, cognitive dysfunction, sickle trait, sickle cell trait

## Abstract

Sickle cell trait and disease are potential risk factors for dementia and cognitive dysfunction in African Americans, as are genetic variants. This illness affects around 300 million people globally. Due to its ability to defend against severe malaria, it represents an evolutionary survival advantage. It has been shown that sickle cell disease and trait are independent risk factors for the prevalence and incidence of albuminuria and chronic renal disease. Sickle cell anemia impairs cognitive performance in people with minimal or mild manifestations of the genetic blood disorder, owing mostly to its cerebrovascular implications. Similarly, various cerebral minor vascular disorders, such as silent cerebral infarcts, have been linked to the sickle cell trait, which is associated with impaired cognitive ability. It has been found that patients with sickle cell disease have a significantly decreased subcortical and cortical brain volume. Adults and children with sickle cell disease have been documented to have attention-related issues, particularly reduced sustained attention.

## Introduction and background

Many vascular risk factors are associated with the decline in cognitive abilities and sometimes complete impairment, intermediated partially by short subclinical strokes [1,2]. Patients with sickle cell anemia can develop cognitive function impairments. It is noted that particularly adults [3] and children [4] having sickle cell anemia score poorly in cognitive tests when compared with controls. Similarly, academic performance is also low in children suffering from sickle cell anemia [5]. 

Adults and children with sickle cell anemia sometimes experience silent cerebral infarction [6]. The measure of silent cerebral infarction is white matter hyperintensities which are associated with weaker neurocognitive results, especially in children [7]. Sickle cell anemia is also connected with mental processing agility independent of silent infarcts but associated with the integrity of white matter defined by MRI [8]. Research on adult sickle cell anemia patients has shown a lower subcortical and cortical volume of the brain when compared with controls, which are further associated with poorer cognitive abilities [9].

Collectively, these results form a hypothesis that sickle cell anemia is a potential risk for the impairment of cognition which can use various possible mechanisms like sickling in smaller vasculature forming obstruction, small subclinical or clinical strokes, and other metabolically occurring disorders such as hypoxia [10], coagulation activation [11], and inflammation [12]. Individuals with sickle cell trait have a raised risk for stroke [13], venous thromboembolism [14], and kidney infarction [15]. Probable mechanisms behind these connections are sickling of cells in smaller vasculature under hypoxic conditions, increased coagulation, inflammation, or other biochemical influences of sickling [11].

This traditional review clarifies the background, etiology, epidemiology, and issues related to sickle cell disease and dementia. It also emphasizes the deteriorating effects of sickle cell disease on the cognitive function of children and adults. The primary purpose of this review is to highlight the potential underlying mechanisms associated with the development of dementia and other cognitive dysfunctions in sickle cell disease.

## Review

Sickle cell trait: An insight into its importance and prevalence

Sickle cell trait is a genetic problem that has about three million cases worldwide [16]. It represents an evolutionary survival advantage due to its protective properties against severe malaria [17]. This trait is highly prevalent in the African-origin population residing across the globe [18]. In the USA, data from newborn screening represent that national estimates of sickle cell trait are around 1.5% and with 8% prevalence in Americans of African origin [19]. In the European population, about 1% to 3% of the population have hemoglobinopathy-associated gene mutation, especially sickle cell trait [20]. A considerable hemoglobin gene variant is present in about 5% of the world [21]. According to a recent estimate (2013) of birth time prevalence of sickle cell disease, about 100 and 12 per 100,000 livebirth have sickle cell disease and is 10 times higher in Africa [18].

Etiology of sickle cell trait

Sickle cell trait exhibits a heterozygous state where red blood cells have one copy of mutant hemoglobin donated as hemoglobin S (HbS) and one copy of normal adult hemoglobin (Hb) i.e. HbA together which produce a genotype of HbAS [22]. An amino acid substitution replacing glutamic acid with valine due to missense mutation produces an HbS state [23]. Although HbS phenotype is attenuated due to the occurrence of HbA which decreases the chances of polymer formation, the majority of the population of sickle cell trait represents normal hematological parameters [23]. However, overall evidence shows that hypoxia due to sickle cell trait can prompt associated sickle issues like exertional rhabdomyolysis, papillary necrosis, thromboembolism, splenic infarction, neurobehavioral changes, and death [24-29].

Cerebral small vasculature diseases and dementia associated with sickle cell trait

Until now, this variant was considered benign in its clinical pattern. Recently, a study demonstrated that the sickle cell trait is an independent risk element in the prevalence and incidence of albuminuria and chronic kidney disease [15]. Moreover, different studies demonstrate that there is a considerably greater risk for cardiovascular phenotypes and atrial fibrillation in sickle cell trait-bearing African Americans [30]. Both atrial fibrillation [31] and chronic kidney disease [32] are found to be considerably linked with a higher risk of dementia and cognitive loss. According to a recent study, young individuals of African ancestry have a considerably higher chance of indicating silent cerebral infarct on MRI when compared with the healthy controlled population (sex and age-matched).

Sickle cell trait describes the inherited one usual l β-globin gene and one sickle β-globin gene. It occurs in about 8% of African Americans, which is about three and half million in number. Homozygousity or double heterozygosity is considered sickle cell disease. Any point mutation at the β-globin gene causes to produce sickle β-globin mutation, which moves a glutamic acid codon (GAG) to a valine codon (GTG) at the sixth position in the chain of β-globin [33]. Many cerebral small vascular diseases such as silent cerebral infarcts are linked with sickle cell trait [6], which in turn is associated with weaker cognitive abilities [7]. Moreover, a considerably lesser subcortical and cortical brain volume in patients with sickle trait has been reported after adjusting the associated risk factors [9].

Neurocognitive impairments in sickle cell trait and disease

Until recently, a large number of studies demonstrated a greater ratio of damage on mental processes in pediatric sickle cell disease in contrast to the common population [34]. Working memory issues seem to be more evident in executive functions [35]. Adults and children having sickle cell traits are reported to have associated attention difficulties and especially impaired sustained attention was prominent in children with sickle trait [36]. Currently, limited literature is available on memory function and its association with sickle cell trait [4]. Intellectual activity impairment can occur in sickle cell trait as about 25% of individuals with sickle cell trait have a significant cognitive issue [37]. In a small sample of patients with sickle trait but without clinical stroke, the incidence of mild mental loss was raised 11-fold. The full-scale intelligence quotient (IQ) of these individuals was also associated with pack cell volume [38]. Sickle cell disease is a long-lasting issue, hence age-associated influences were also studied. Cross-sectional studies demonstrate that the neurocognitive issue of sustained attention, spatial functioning, and reading achievement was higher in older children [39]. In various vaso-obstructive conditions at the micro-vascular level, hypoxemia is illustrated as a key precipitating factor and also contributed to silent ischemic injuries at the cerebrovascular level, which are responsible for various neurocognitive dysfunctions such as long term, and short term memory loss, lack of executive function, impaired attention, and learning problems [40]. Many studies highlighted that children with sickle cell disease represented defective autoregulation of cerebral blood flow when compared with healthy children irrespective of their hemolysis rate [41]. That is why impaired cognitive function in sickle cell trait patients is thought to be due to chronic hypoxia of the brain [42]. Children with sickle cell trait and evident strokes are commonly represented with neuropsychological complications that are associated with the size and location of the lesion in nervous tissue [43]. Furthermore, other identified dysfunction areas can be learning problems in mathematics and reading abilities [44]. In children who experienced silent infarctions, intellectual functionality was also documented in association with the size of the lesion [45]. Though there are limited studies available on neuropsychological problems associated with sickle cell traits in adults, impaired cognitive functions such as dementia have been described independently of abnormal or normal results of MRI [46]. Proof from children having sickle cell disease proposes that the size of the lesion and associated neuropsychological problems inclines to increase with age [47], which could present the same issues in adult patients. Additionally, abnormal blood flow toward the frontal lobe has been demonstrated in adults having sickle cell disease [48], which points to executive function and concentration/attention issues. Thus, it cannot be ignored in hematological studies [49].

Neurological sequelae of sickle cell trait and disease

The association of memory loss issues in children having sickle cell trait is not known yet. Different factors have been attributed in the association of these two conditions, i.e., cognitive and academic performance can be compromised due to concurrent hospitalizations due to chronic illness, indirect influence of environment, and social influence [50]. However, the sickle cell trait influences the blood supply in the cerebrum that can result in infarction, especially in the frontal lobe of the brain [51]. Cerebral infarction has been assigned as an acute neurologic condition resulting from occlusions of small hemorrhages in the brain vasculature leading to nervous signs and symptoms that may last longer than 24 hours [52]. Moser et al. [53] investigated that children having sickle cell disease experience brain infarction before they are 14 years old. Fourteen percent of the children having sickle cell disease experience silent brain infarction before they are 14 years old (the average age for the onset of this condition is six years). The incidence or onset of clinical infarction is 14 times higher in patients with silent infarction. The impaired cognitive performance in sickle cell trait has been connected to multiple findings in different domains. The advances in neurology have made it more convenient to differentiate between silent infarction, clinical infarction, or no infarction in cerebral neuroimaging [54].

Effect of sickle cell trait on general intelligence (IQ)

The intellectual skills or performance of an individual suffering from sickle cell disease have been surprisingly impaired and compromised in multiple degrees. The full-scale IQ level (FSIQ), performance IQ level (PIQ), verbal IQ level (VIQ), or all these in combination can be impaired in sickle cell disease-induced neurological changes. However, a few studies did not find any significant influence between the two conditions [55, 56].

The IQ level can be measured at the age of two and a half years in children. The poor performance of the children having sickle cell disease has been evidenced compared to controlled groups of similar age and ethnicity in Bayley Infant Neurodevelopmental screener [57]. There is a delay in the development of the brain and nervous tissues in children with sickle cell disease that is attributed to increased cerebral blood flow velocity in middle cerebral, internal carotid, and basilar arteries at the age of nine months. Wang et al. (1998) reported the evidence of subclinical leukomalacia, stenosis, and tortuosity in children having sickle cell disease in neuroimaging findings. Children with these neurological abnormalities showed cognitive impairments due to compromised nervous tissue development. The IQ level of these children is also compromised, deepening upon the degree of neurological impairment. Infarction, either clinical or subclinical, and hypoxia is a consequence of sickle cell disease that is the leading factor for cerebral vasculature damage that results in lower performance, verbal, and full-scale IQ levels [47]. Several studies have been conducted to test the IQ levels of children with sickle cell trait with subclinical, clinical, and no infarction. A significant difference in the verbal, performance, and full-scale IQ level of the children with clinical and subclinical infarction has been observed as compared to children with no infarction [44, 47]. Many children with subclinical infarction were found to have compromised performance in terms of verbal and full-scale IQ level as compared to those with no infarction. And lowered scores in performance IQ were also observed in the same study groups (the group with silent infarction vs no infarction) [44, 47]. In the same study, children with clinical infarction were compared with children having subclinical infarction. A significant difference was observed between these two groups. Children with clinical infarction were deficient in performance and full-scale IQ level. The children with clinical, subclinical, and no infarction were also compared in another study with healthy siblings. The lowest score of the three types of IQ was observed in children with clinical infarction as compared to other groups [58]. 

Different methods can be used to measure the neurological abnormalities and compromise depending upon different types of criteria, and the association between neurological abnormalities and cognitive function can vary accordingly. The children identified with infarction via CT were evidenced with very poor IQ levels as compared to children with no infarction [59]. Bernaudin et al. demonstrated the relation of low IQ score with neurological disorders and abnormalities measured in transcranial doppler (TCD) and MRI [60]. The study found a remarkable deficiency in performance and full-scale IQ of the children with clinical infarction as compared to children with no infarction. Verbal and full-scale IQ scores were compromised in children showing abnormalities in their MRI scans as compared to children with normal MRI scans.

Kral et al. did not observe any deficiency in performance scores of the two groups compared, but they did evidence poor verbal IQ of the children with abnormal TCD scan in contrast to the control group on TCD [61]. However, the distinction in the full-scale IQ score was not evidence-based upon TCD scans. The children having sickle cell trait may also develop MoyaMoya syndrome, in which there is a progressive formation of several tiny collateral vessels that leads towards stenosis in the internal carotid arteries. Such children with sickle cell trait and evidence of brain infarction and MoyaMoya syndrome exhibited more cognitive impairment in terms of verbal IQ level as compared to children with sickle cell trait but no MoyaMoya syndrome, children with no sickle cell trait but MoyaMoya syndrome, and children with no sickle cell and MoyaMoya syndrome (healthy group) [57].

The damage to nervous tissue has a detrimental effect on the cognitive abilities of a person. Steen et al. organized the children with sickle cell disease into different groups depending upon the ventricular volume and brain lesion [45]. A deficiency in full-scale IQ level of children with higher lesion and ventricular volume was found as compared to children with a lesser number of lesions and children showing no abnormality on MRI. 

The site of the infarction in the brain can also affect the degree of IQ level. Children with infarction on the left cortical were deficient in verbal, performance, and full-scale IQ level compared to the study norms. In children with right side infarction, the brain cortical showed deficiency in performance and full-scale IQ level but no difference in verbal IQ was noticed. The study showed that left side cortical infarction could result in more impairment of the IQ levels as compared to right side cortical infarction [62].

As evidenced by many studies, it is a fact that infarction influences cognitive performance. Many studies have shown cognitive impairment in children having sickle cell disease but no cerebrovascular damage. Steen et al. investigated remarkable differences in verbal, performance, and full-scale IQ levels of children having sickle cell disease but normal MRI scans [63]. However, the use of quantitative MRI scans in children with sickle cell disease who showed normal scans and imaging on conventional MRI exhibited the proof of very minute changes at the cellular level [64]. The children with sickle cell trait, with or without abnormalities in the brain or nervous tissue, revealed a marked decrease in the full-scale IQ level compared to the healthy control group of the same age and ethnicity, and the children having sickle cell trait exhibiting neurological abnormalities showed a more significant deficiency as compared to children having sickle cell trait but no neurological abnormality. 

The conclusion of many studies has investigated the poor intellectual performance of children with infarction compared to children who did not reveal any neurological abnormality. Mixed results were found in children for performance IQ levels in study groups having children with silent and clinical infarction. Even without any damage to cerebral vasculature, many children with sickle cell traits showed poor performance in different IQ measures compared to healthy control groups. Other than intellectual ability, other domains of life can also become affected due to the presence of sickle cell traits. 

Effect of sickle cell trait on memory

Very low evidence regarding the degree of memory function due to sickle cell trait is available in the literature. Only one study out of seven conducted a comparative study in three groups of children: one with clinical infarction, the second with silent infarct, and the third group with no infarction for the assessment of their memory functioning. Visual memory deficits were found in children with clinical infarct as compared to other groups studied. In the context of verbal memory, children with clinical infarct were compromised on paired task associates, but no differences were found in terms of prose material in all the groups [58]. On the contrary, there was no difference observed in the memory performance of the children with silent infarct compared to children without sickle cell disease and children having sickle cell disease but with no brain infarct [4,58].

Cohen et al. explored the relationship between the memory and location of infarct lesions in children with sickle cell disease [62]. It was demonstrated that children with left side cortical lesions were compromised in visual-spatial memory and auditory-verbal memory. Those having right-side cortical lesions were deficient in visual-spatial memory only. Both short and long-delayed free recall impairment was observed in children with infarct in the anterior lobe compared to children having sickle cell trait without infarction [35]. The three groups studied did not reveal any difference in long or shot belated cued or recognition call.

Limitations of literature available

In the domains of executive function and attention, memory seems to be less prominent or least studied. Neurological integrity is greatly associated with memory function. Impaired cognitive functions such as dementia have been described independently of abnormal or normal results of MRI. Proof from children having sickle cell disease proposes that the size of the lesion and associated neuropsychological problems are inclined to increase with age [47], which could present the same issues in adult patients. Additionally, abnormal blood flow toward the frontal lobe has been demonstrated in adult people having sickle cell disease [48], which can point out executive function and concentration/attention issues, thus, hematological studies should not be ignored. However, there are limited studies available on neuropsychological problems associated with sickle cell traits in adults. Similarly, studies examining memory function in association with neurological damage in sickle cell traits are limited. It is a fact that impaired cognitive development and memory issues can greatly impact the quality of life in individuals with sickle cell trait. This traditional review emphasizes the dire need for further investigations on the impact of sickle cell trait on neurological integrity, neurocognition, and memory function to better understand the underlying pathways in the development of these issues. 

## Conclusions

Sickle cell disease and trait are genetic abnormalities that may increase the risk of dementia and cognitive dysfunction, especially in African Americans. Sickle cell trait was not found to be an independent risk factor for prevalent or incident cognitive decline, but it may interact with and affect other risk factors for dementia and cognitive decline. Sickle cell trait is also linked to albuminuria and chronic renal illness, as well as cardiovascular phenotypes and atrial fibrillation. Both atrial fibrillation and chronic renal disease are associated with an increased risk of dementia and cognitive decline. Furthermore, different cerebral minor vascular disorders, such as silent cerebral infarcts are linked with sickle cell trait, which is associated with weaker cognitive capacities. Also, the risk of stroke is highest in individuals with sickle cell disease, which in turn leads to cognitive dysfunction. Attention deficits, particularly poor sustained attention, were common in children with sickle cell trait. However, there is a paucity of research on memory function and its relationship to sickle cell trait.
